# Supplementary figures and images for: Protein methyltransferase 7 deficiency in Leishmania major increases neutrophil associated pathology in murine model
Source: PLoS Negl Trop Dis. 2021 Mar 2;15(3):e0009230. doi: 10.1371/journal.pntd.0009230 (PMC7954300; doi:10.1371/journal.pntd.0009230)

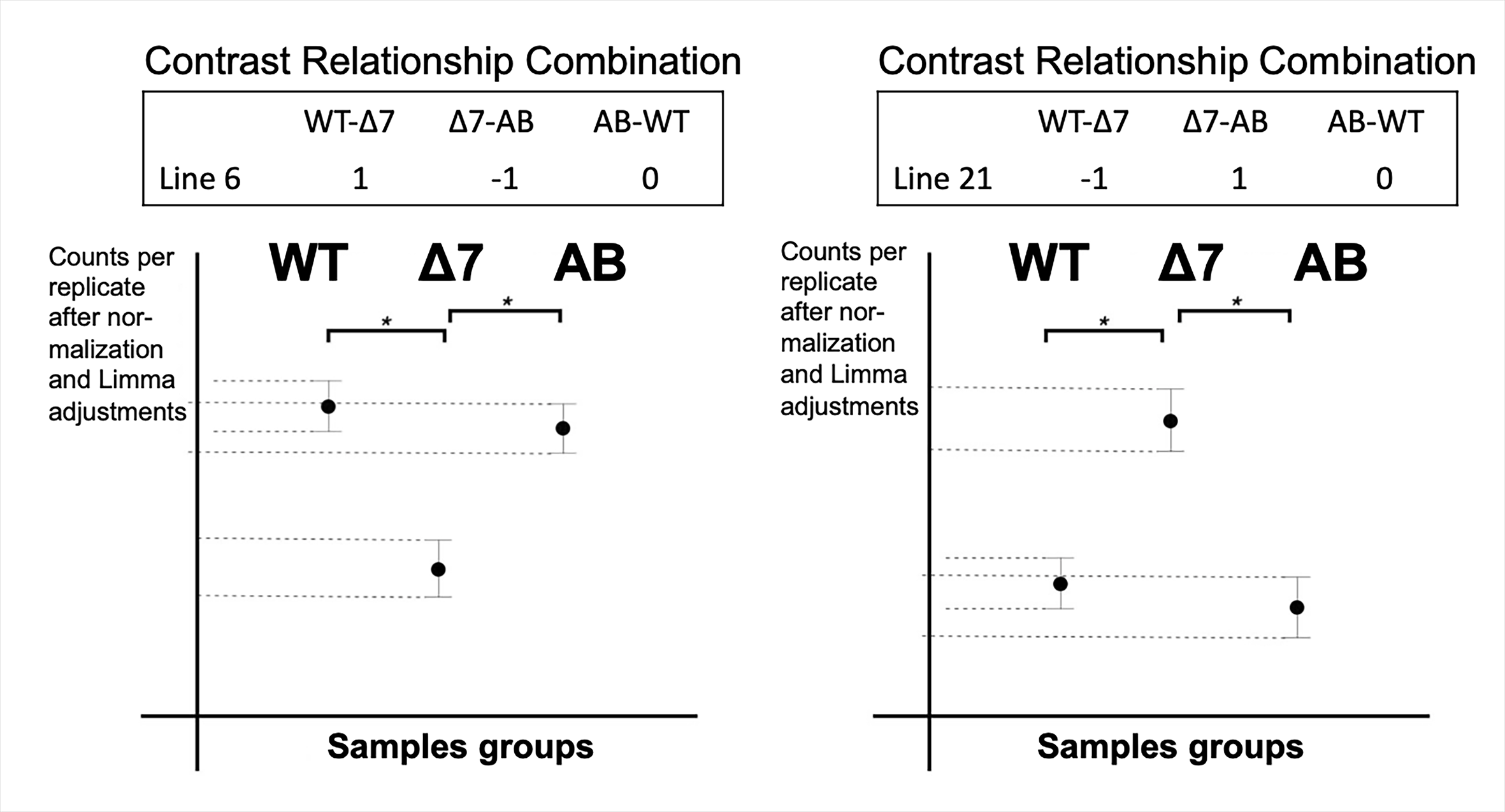

Supplement: S1 Fig — This scenario depicts only those genes differentially expressed in Δlmjprmt7 (Δ7) samples compared with wild type (WT) and Δlmjprmt7 [PRMT7] (AB). Genes expressed at different levels in WT and AB samples were omitted. Down-regulated genes are shown on the left panel and upregulated on the right. (TIFF) [file pntd.0009230.s001.tiff]

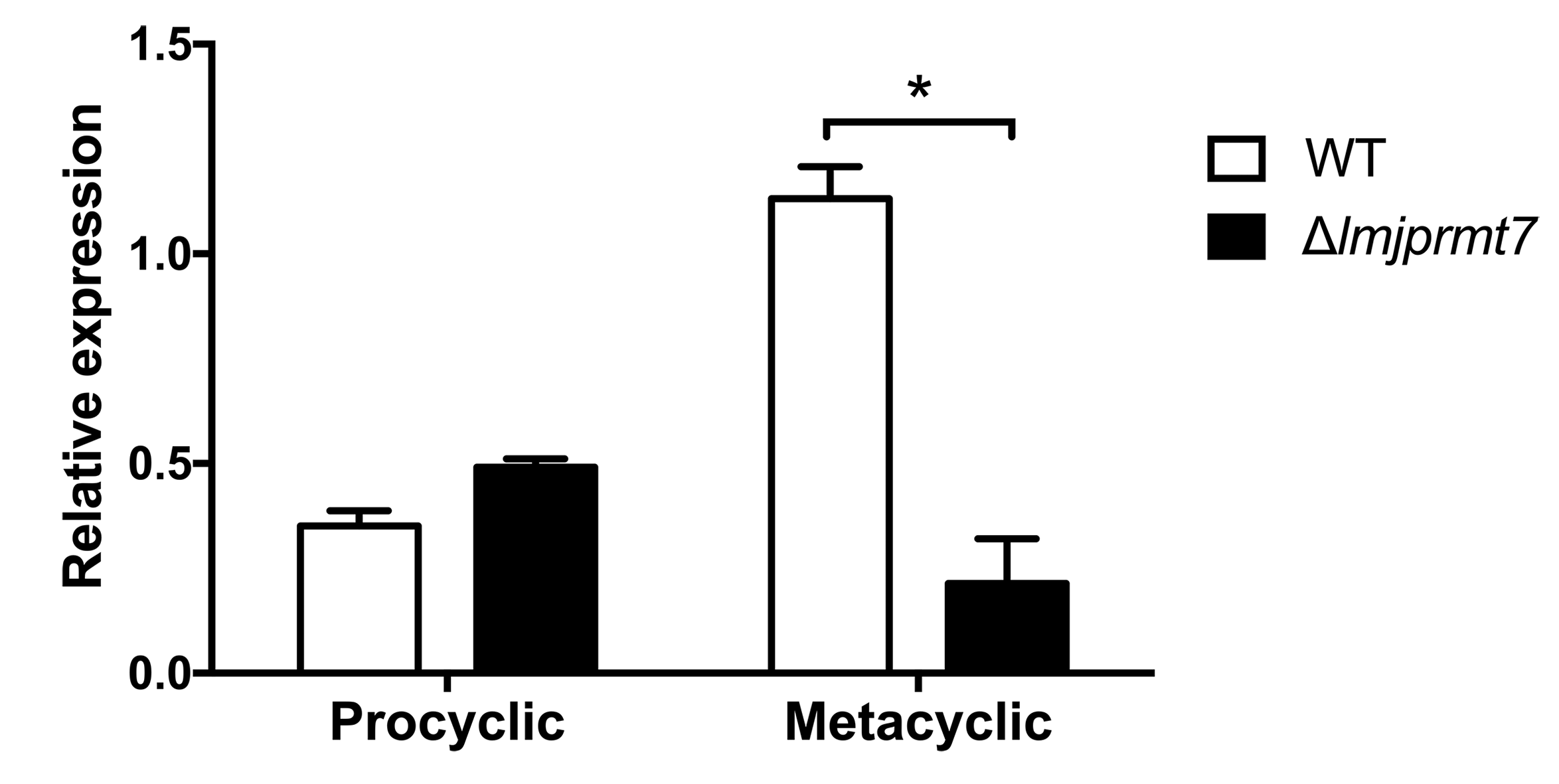

Supplement: S2 Fig — Quantitative RT-PCR analysis of RNA extracted from promastigotes during the early-log (procyclic) and purified metacyclic phases. Comparing L. major CC1 WT and Δlmjprmt7 strains. The expression is relative to the expression of the G6PD and RNA45 genes. Statistical analysis was performed by Two-way ANOVA followed by Sidak post-test. (TIF) [file pntd.0009230.s002.tif]

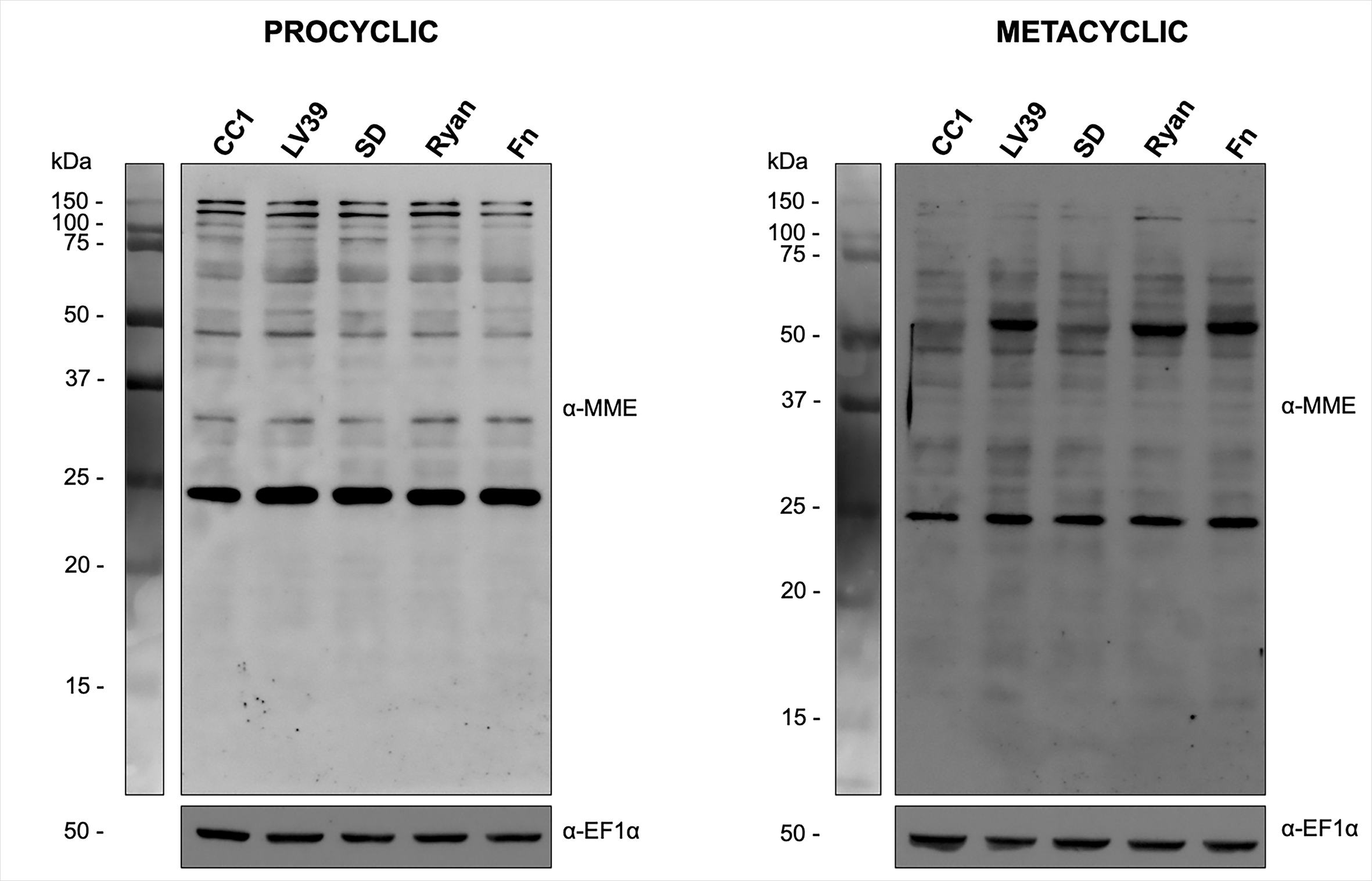

Supplement: S3 Fig — Arginine monomethyladed levels in procyclic (left panel) and enriched metacyclic (right panel) promastigotes. The pathogenic Leishmania major strains: Ryan, LV39, Sd and Friedlin (Fn) and the nonpathogenic CC1 strain were incubated with the anti-monomethylarginine antibody (α-MME) overnight at 4°C. The lower panels show loading control with an antibody against the Elongation factor 1-α, as indicated (α-EF1α). (TIFF) [file pntd.0009230.s003.tiff]

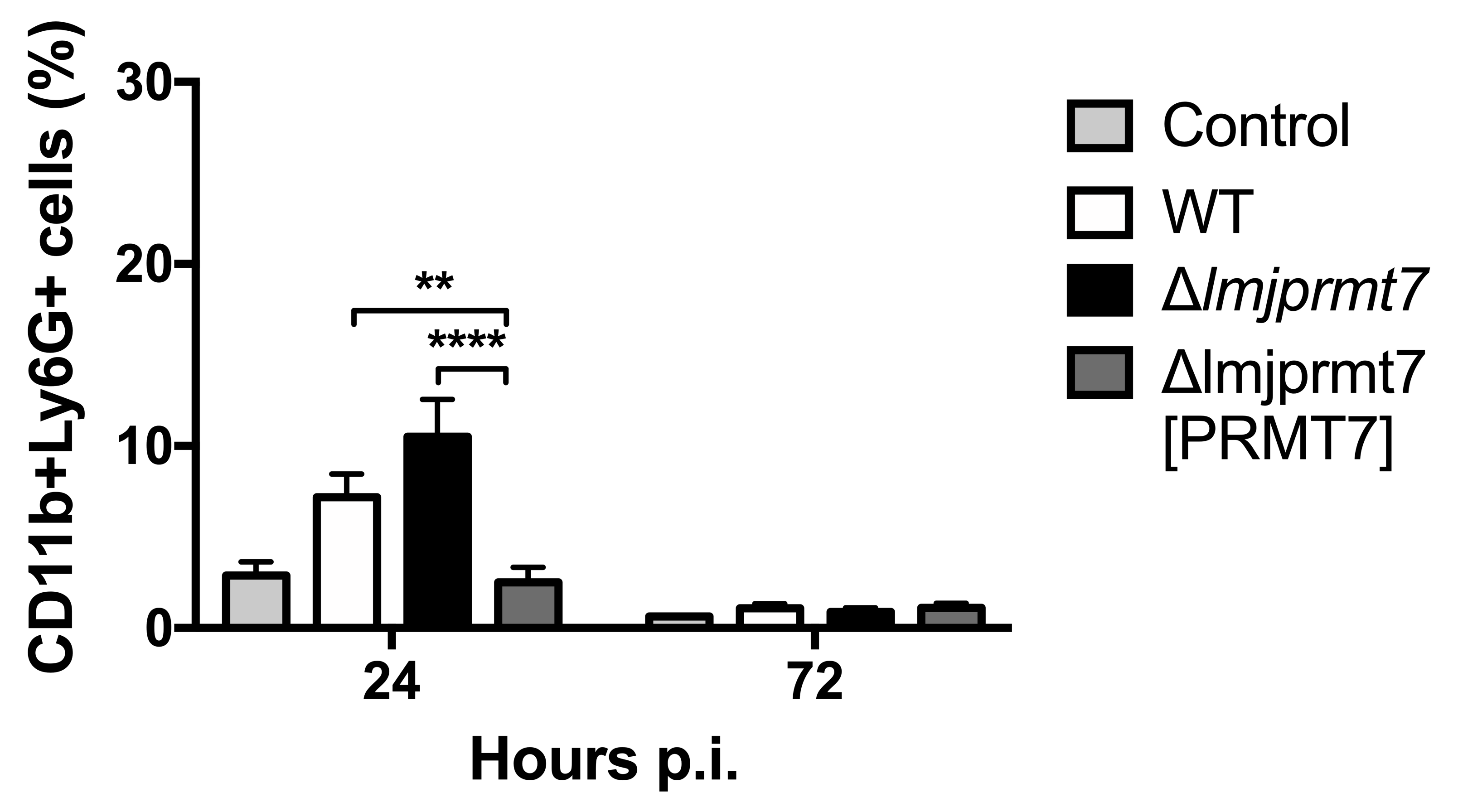

Supplement: S4 Fig — BALB/c mice were infected in the ear dermis with 100,000 Lmj CC1 metacyclic promastigotes from the wild type, Δlmjprmt7 and Δlmjprmt7 [PRMT7] strains. Ear tissues were processed at 24- and 72-hours post-infection to study the cell recruitment by flow cytometry. All data are shown as the mean ± SEM of four samples per group. Representative data from two independent experiments. ** p<0.01 and **** p<0.0001, using two-way ANOVA followed by Tukey’s multiple comparison test. (TIFF) [file pntd.0009230.s004.tiff]
