# Supplementary material for: Protein methyltransferase 7 deficiency in Leishmania major increases neutrophil associated pathology in murine model
Source: PLoS Negl Trop Dis. 2021 Mar 2;15(3):e0009230. doi: 10.1371/journal.pntd.0009230 (PMC7954300; doi:10.1371/journal.pntd.0009230)
Supplement: Attachment — Submitted filename: 201230_Response_Reviewers_AlcoforadoDiniz.docx [file pntd.0009230.s008.docx]

--------------------

RESPONSE TO REVIEWERS - PNTD-D-20-01508

*Protein methyltransferase 7 deficiency in Leishmania major increases neutrophil associated pathology in murine model*

Reviewer #1: Ferreira et al. report on their progress in characterizing the effect of protein methyltransferase 7 on parasite fitness and virulence throughout the life cycle in Leishmania major. This is a logic extension of their previous work but also a formidable task given that one expects a polyphenic phenotype in Lmprmt7 deficient parasites derived by genetic manipulation (as originally described in ref 13).

The strengths of the work are related to the characterization of parasites’ phenotype in the major habitats encountered during the life cycle. In the sandfly vector quantitative development is severely affected and ‘metacyclic’ parasites morphs seem to be severely reduced if parasites are Lmprmt7 deficient. In contrast, lesion development in mice seems exacerbated and this correlates with an increased local presence of neutrophils. The experiments and approach to characterize these phenotypes are appropriate and well conducted.

The major weakness in view of this referee is related to the interpretation of the results which is not straight forward because of inconsistencies with the previously published findings using the same parasites (ref. 13). A major issue is the apparent dramatic regain of pathogenicity with respect to lesion induction of the Lmprmt7 deficient strain which presumably is still the same as the one described in 2014. The previously published data (Fig. 4b in Ferreira et al.2014, ref 13) suggest a very minor gain of pathogenic potential by deleting Lmprmt7. This has apparently changed dramatically since (Fig. 3A this work).

We thank the reviewer for the careful analysis of the manuscript. The inconsistencies are only apparent as we may explain;

Herein we used intradermal injection in mice ears and in the previous work this was done sub-cutaneous in the mice footpad. In addition, we now injected a fraction of stationary phase parasites which was enriched for metacyclic promastigotes (Ficoll gradient), and this represents a major difference in terms of efficiency of infection. A third aspect is that despite the absolute lesion size differences observed in Ferreira and Diniz studies, the fold differences within each study between lesions induced by Wild Type and PRMT7 KO lines are similar.

It is noteworthy that, according to the authors, Lmprmt7 could not be deleted inLV39 another parasite isolate. This indicates on the one hand an essential role of the gene depending on genetic background, and, on the other hand, a strong selective pressure for compensating mechanisms to evolve to simply maintain viability. Thus, parasites originally described in 2014 may have accumulated compensatory mechanisms and this is not considered in the present work. In particular, should the strains have been passaged since several times in vivo which is a common routine in many labs working with L. major to maintain the virulent phenotype because that can be prone to lose it if only passaged in vitro. Thus, a simple approach to control for this is to re-complement the currently used Lmprmt7 deficient strain.

After the Ferreira study, we established the CRISPR/Cas9 system for transfecting the parasites in the lab. Using this editing tool, we have successfully deleted the PRMT7 gene from the LV39 strain. Thus, the previous failure was probably due to the lower efficiency of the classical homologous recombination protocol as compared to CRISPR/Cas9. PRMT7 KO was also achieved in *L. braziliensis* (Lorenzon et al, in preparation). Therefore, there is no reason to believe that compensatory mechanisms explain the results. In addition, we do not maintain *Leishmania* lines under long-term serial passages to avoid loss of ‘virulence’. In addition, the addback strain, as shown by the exemplary recovery of the wildtype phenotype, corroborates our hypothesis regarding the role of PRMT7. With respect to this point, we present below a PCR result confirming that the addback line contains the PRMT7 gene.

A similar point of inconsistency over time is the behaviour of the L. major CC1 strains in vitro in macrophages where differences were claimed to emanate from Lmprmt7 deficiency (Fig. 6c this ms and Fig. 4a ref 13).

We took into consideration the comment and after carefully reanalyzing our data we have decided to exclude this result. In addition, we performed an *in vivo* analysis of the uptake by macrophages and it also corroborates with Ferreira’s previous data (404-408, and revised figure 6).

Finally, Lmprmt7 may have moonlighting functions. Hence, complementation with a functional null mutant may also be worth considering.

The reviewer raised a very interesting hypothesis that we cannot ignore. Therefore, we modified the text (lines 578-581) to make it clear that moonlighting activity might occur in PRMT7, and we do not have the ultimate proof that the methyltransferase activity of PRMT7 would be at the center of the phenotypic alterations we observed.

Minor points:

1. If indeed neutrophil response is functionally linked to Lmprmt7 presence then authors may contemplate using nude or rag-deficient mice that would eliminate the immunopathological component of the T cell response that is most prominent in BALB/c mice.

Although an interesting suggestion, we would argue that the proposed experiments would not be so informative since both T cells and neutrophils might be required for the pathology. We emphasize that we have already investigated the infection in two different mouse genetic backgrounds, BALB/c and C57BL/6.

2. The complemented strains are described as having ectopic copies of Lmprmt7 gene which is a sort of vague description given that episomally and genomically compensated strains were constructed originally. This should be specified as it further affects interpretation and review of the results.

The vector pX63Neo (LeBowitz et al., 1993) was chosen to generate the PRMT7 addback parasite. The insert contained the original copy of lmjPRMT7 with the corresponding UTRs (800 bp upstream and 800 downstream). The circular construct was transfected in the lmjPRMT7KO parasites. The presence of the insert has been confirmed by PCR, the parasites are G418 resistant and we have a PFGE southern blotting result which shows that the circular construct is present. While integration might have occurred, which we did not further examine this possibility. We understand that important in this context is the fact that the endogenous UTRs were kept to control PRMT7 gene expression. We have not added this information in the revised version of the manuscript, since this strain has previously been published (Ferreira et al., 2014).

PFGE: The gel depicted on the left side contains the karyotype of the indicated lines/transfectants and it was transferred for a southern blotting analysis with a PRMT7 fragment as a probe.

PCR showing the *LmjPRMT7* gene present in the WT and addback strain. The primers used were:

Forward: GATCTGGTTGATTCTGCCAGTAG

Reverse: GTACATCTCGAGACCACCGCTACGGTGGACGGC

3. Quantitative real time PCR to compare Lmprmt7 mRNA levels is suboptimal given the mentioned reliance of gene expression on post-transcriptional mechanisms, protein and, more relevant, activity would be much more convincing. Moreover, the choice of a pentose-phosphate pathway reference or housekeeping gene can be misleading when comparing life cycle stages since the pathway is clearly regulated as well (e.g. Rosenzweig et al.2007 for the situation in *L. donovani* which applies to other species too).

As suggested by the reviewer we analyzed the monomethylation profile in the distinct L. major strains by western blotting. The result, added as supplementary material (S3 Fig), indicates no major differences on the monomethylation levels when comparing the attenuated line with the pathogenic lines used. The corresponding text has been modified (lines 311-312 and 578-581).

In addition, we included in the revised version a RT-qPCR with two different endogenous genes (new figure 2A).

Reviewer #2: In this manuscript, Diniz et al report that one of Leishmania major protein methyltransferases, LmjPRMT7 has an impact on parasite virulence *in vivo*. High levels of LmjPRMT7 can impair the pathogenicity observed following infection of wild type mice. This was shown by restoration of virulence in an attenuated L. major strain upon LmjPRMT7 deletion. Although a significant impact was observed on lesion development following infection with *L. major* parasites deleted of *LmjPRMT7*, no impact was observed on parasite control 7 weeks after infection. In contrast, the absence of *LmjPRMT7* impaired parasite development in the sand fly vector. Putative genes modulated by the absence of this enzyme are presented. The observations are clearly presented.

We thank the reviewer for the comments and suggestions that contributed to improving the manuscript.

Specific comments to the authors:

Major comments:

1) The authors conclude (p.23) that neutrophils rather than macrophages play a major role in the uptake of parasites lacking LmjPRMT7. However, 4 hours post infection, macrophages take up 35 % of parasites (with or withoutLmjPRMT7) while neutrophils uptake is of approximately 40% for LmjPRMT7-deleted parasites. Thus, the difference between macrophages and neutrophils does not appear to be a major one. a possible difference in parasite uptake by macrophages and neutrophils should be confirmed in vivo by flow cytometry analysis.

Following the reviewer suggestion, we analyzed the uptake in vivo and the new experiment modified the outcome. Regarding neutrophils uptake, we observed that the *in vivo* results do not correlate with the *in vitro* ones. In the revised manuscript, we kept the *in vitro* result for neutrophils showing the higher uptake of the parasites lacking the enzyme in the main text and in the supplementary material we added the *in vivo* flow cytometry analysis of mCherry fluorescent strains. Again, based on the reviewers comments we checked the in vivo uptake by macrophages and observed a higher uptake in the knockout parasites similarly to what has been observed previously *in vitro* (Ferreira, 2014). The *in vivo* result is shown in the supplementary material. The corresponding text has been modified in the manuscript (404-408) and the *in vivo* uptake results are shown in the revised Fig 6C.

2) The main phenotype observed in mice infected with ΔPRMT7 parasites is the development of a necrotic lesion associated with the presence of neutrophils. The author hypothesize that sustained neutrophil recruitment to the site of infection is responsible for exacerbated disease (p. 21). To assess if increased migration of neutrophils is responsible for the bigger lesion observed, the authors should first investigate if increased neutrophil recruitment is observed in the first days (24 hours and 72 hours) post infection, two time points where neutrophils were shown to be recruited or not, respectively, to determine an impact of the lack of PRMT7 on neutrophil migration per se. Transwell experiments could also be performed to compare the neutrophil recruitment by the different strains

As suggested by the reviewer we analyzed the neutrophil recruitment in the early infection time points (24h and 72h p.i.). Indeed, there is no difference between the KO and control strains, since all strains show a low neutrophil recruitment rate in this early time points. These results were added in the text of the revised version (lines 354-367) and as a supplementary figure (S4_Fig).

3) Figure 5: It is clear that the number of parasites per ear does not vary between WT, ΔLmjPRMT7 and the add back control. What is more surprising is that the number of parasites observed in the ears of C57BL/6 mice is quite elevated for mice of a resistant genetic background with values within the range observed in lesion of infected BALB/c mice. Could the authors comment on this.

Certainly, the parasite burden does not vary among the strains in the two different mice genetic background tested. Regarding the number of parasites in the C57 resistant background, we want to call the attention to the fact that the Y axis is expressed in -log_10_ scale. The C57BL/6 mice present 1 to 2-fold less parasites than the BALB/c mice. We checked parasite titers in articles using a similar approach and found values varying from 4 to 7-log_10_ when infecting C57BL/6 mice (refs 33-35, cited in the manuscript).

4) The impaired formation of metacyclic ΔLmjPRMT7 in the sand fly should be discussed further

We did not mean to affirm that there was an impairment in the metacyclogenesis in the KO strain compared with control lines in the sandfly. Instead, we have shown that the parasite load was impaired in sandflies infected with the KO strain. We sought to perform RT-qPCR with SHERP, a marker for metacyclogenesis, in the infected sandflies. Nevertheless, we recovered no detectable levels of the parasite RNA. Preliminary morphological analysis of the gut smears indicated a very small percentage of metacyclic in general, in parental (CC1) and transfectant lines, but the Covid19 pandemic suspended the use of the microscopy facility. Thus, we cannot make any statement on the effects of PRMT7 on the metacyclogenesis in the sandfly. The text was modified in the revised manuscript (Line 482).

Reviewer #2: Minor comments:

1)Figure 2. Higher levels of PRMT7 mRNA are observed in L. major CC1, a non-pathogenic strain of L. major. Have the authors any data showing that there is a correlation between PRMT7 mRNA levels and the activity of the enzyme. This could lead to possible differences in PRMT7-dependent methylation in the different L. major strains with consequence on the PRMT7 gene control at post transcriptional levels. This should at least be discussed.

As suggested by the reviewer we analyzed the monomethylation profile in the distinct L. major strains by western blotting. The result, added as supplementary material (S3 Fig), indicates no differences in the monomethylation levels among the lines used. The corresponding text has been modified (lines 311-312 and 578-581).

2) The CC1 L. major strain is attenuated however, it is not correct to state that “L. major CC1 did not cause any lesion” (p. 14 line 287). Figure 2C clearly shows the presence of a lesion which is smaller than those observed following infection with the other strains but clearly present. Please modify the text accordingly.

As suggested, we modified the corresponding text (line 315)

3) Although the authors state that they use the ear model of infection involving intradermal infection with a low number of parasites (p. 19) they infect mice with a high dose of parasites (10e5). This should be corrected.

As suggested by the reviewer, the text has been modified accordingly (lines 492).

4) Figure 2D ** is significant compared to what?

For the parasite burden the statistical analysis was conducted comparing virulent strains with the *Lmj*CC1 attenuated line. This information has been added to the legend of Fig 2 (lines 772-773)

5) The authors conclude (p.22) that their data provide a novel link between neutrophil mediated inflammation and lesion formation. The correlation between neutrophil presence in unhealing lesions and lesion formation has already been reported by several groups (complete references). The novelty is the involvement of LmjPRMT7 in this process even if, whether neutrophils are the cause, or the consequence remains to be determined.

We agree with the reviewer’s comment and modified the text accordingly (line 575).

6) PRMT7 is acting mostly post-transcriptionally thus it is not so surprising that only few differentially expressed transcripts were detected between LmjPEMT7KO parasites and WT strains. The authors have performed proteomics on this parasite strain, together with the present data this will lead to further studies.

We agree that proteomics information will better inform the roles of PRMTs.

7) Typos in supplementary figure 1.

We modified the text of S1 figure for the sake of clarity (lines 844-847) and corrected the typo in figure S1.
