# Supplementary material for: Protein methyltransferase 7 deficiency in Leishmania major increases neutrophil associated pathology in murine model
Source: PLoS Negl Trop Dis. 2021 Mar 2;15(3):e0009230. doi: 10.1371/journal.pntd.0009230 (PMC7954300; doi:10.1371/journal.pntd.0009230)
Supplement: Attachment — Submitted filename: 210201_Response_Reviewers_2nd_Round.docx [file pntd.0009230.s009.docx]

­­Reviewer's Responses to Questions

Methods

Reviewer #1: (No Response)

Reviewer #2: The methodology is appropriate. 
The authors should specify if "control" mice are mice uninfected or mice injected with saline or PBS.

*The control mice were uninfected animals. This information was added in the methodology section, line 134.*

--------------------

Results

Reviewer #1: (No Response)

Reviewer #2: The authors have answered many of the queries.

*We thank the reviewer for the constructive criticism that helped to improve the manuscript*

However, there remains  few points that should be better presented and discussed. 
In the Figure 6C the new data obtained for the ear macrophages are presented but not those for the ear neutrophils. These should be included, as indicated in corresponding  Figure legend, even if they are not similar than waht is observed in vitro.

*We apologize but the revised version of figure 6 was incorrectly uploaded. Figure 6 has been replaced and Fig6C includes both neutrophils (left) and macrophages (right)* in vivo *uptake of tagged* L. major *strains, as described in the figure legend.*

1. In the new Figure S4, the authors state that "there is no difference between the control and KO strains, since all L. major strains used show a low neutrophil recruitment rate in this early time points". The data clearly show a statistically significant difference in neutrophil recruitment in the ear dermis 24 hours p.i. between delta lmjprmt7 and the add back strain (denoted by stars) which show increased neutrophil recruitment. The increase observed between WT and deltalmjprmt7 does not appear significant in the experiment presented, the authors may indicate it this was the case in the other experiment performed.

*The reviewer is correct; we observed an increase in the recruitment of neutrophils by the mutant line compared with the addback strains but not with the wild type. We addressed this observation in the text “We observed a transient initial wave of neutrophil recruitment with a slight increase in neutrophil numbers in the knockout line after 24h. . .” (lines 334-336). Nevertheless, we did not consider this phenotype strong enough maintaining the consistency throughout the manuscript; only those differences observed between the mutant and both control lines (WT and addback strains, simultaneously) were considered a true phenotypic difference.*

The authors should include in the "Discussion" that increased neutrophil recruitment at the onset of infection may have an impact on subsequent development of the disease, as reported by several groups, and may contribute in part to the pathology observed following infection with deltalmjprmt7 mice.

*This is indeed our main conclusion, and we insisted on this point, mentioning the central role of neutrophil recruitment in the discussion section. We ask the reviewer to address lines: 398-400, 467-470, and 509-511.*

Furthermore, while a difference in the frequency of cells was 
observed between deltalmjpmt7 and the add back strain, the difference in neutrophil number observed 7 weeks post infection (Fig.4) was not statistically significant between the  ears of  delta lmjprmt7 and the add back strain, suggesting that the differences observed in the cutaneous pathology may only partially correlate with neutrophil presence. This could be indicated in a few words.

*It is correct; in figure 4B, different subsets of immune cell lines were analysed, and the mutant shows an increase in neutrophil recruitment only when compared with WT cells. For this reason, we repeated the recruitment of neutrophils (after 7 weeks of infection) in two independent experiments. The result of these assays are shown in Figure 4D, and we clearly see the increase in neutrophil recruitment by the mutant when compared with the WT and addback strains (commented on the text, lines 325-331).*

The references cited (33-35) are not using L. major the authors should also include references including L. major that would be more relevant to this study.

*We accepted the suggestion and included two publications using L. major that show the same correlation. The references are included in line 437.*

Conclusions

Reviewer #1: (No Response)

Reviewer #2: As mentioned above, the study is well presented and the results interesting. A few points should be included in the discussion, as suggested above.

--------------------

Editorial and Data Presentation Modifications

Reviewer #1: (No Response)

Reviewer #2: See comments under  the Results section.

--------------------

Summary and General Comments

Reviewer #1: (No Response)

Reviewer #2: The authors have answered most of the querries. Insertion of the the suggestions will complete the study.
